# Spatiotemporal Analysis of Men Who Have Sex With Men in Mainland China: Social App Capture-Recapture Method

**DOI:** 10.2196/14800

**Published:** 2020-01-24

**Authors:** Maogui Hu, Chengdong Xu, Jinfeng Wang

**Affiliations:** 1 State Key Laboratory of Resources and Environmental Information System Institute of Geographic Sciences and Natural Resources Research Chinese Academy of Sciences Beijing China

**Keywords:** HIV risk, men who have sex with men, MSM distribution, migration

## Abstract

**Background:**

In China, the cases of newly diagnosed HIV/AIDS in men who have sex with men (MSM) have increased more than tenfold since 2006. However, the MSM population size, geographical distribution, and migration patterns are largely unknown.

**Objective:**

Our aim is to estimate the number, spatial distribution, and migration of MSM populations in mainland China using big data from social networking.

**Methods:**

We collected 85 days of data on online users of a social networking MSM app in mainland China. Daily online MSM users and their migration across the country were investigated during a holiday period and a nonholiday period. Using the capture-mark-recapture model, we designed an experiment consisting of two independent samples to estimate the total provincial MSM population.

**Results:**

The estimate of MSM in mainland China was 8,288,536 (95% CI 8,274,931-8,302,141), accounting for 1.732% (95% CI 1.729%-1.734%) of adult men aged 18 to 64 years. The average daily number of MSM social networking online across mainland China was 1,198,682 during the nonholiday period. The five provinces (including municipalities) with the highest average number of daily online MSM numbers were Guangdong (n=141,712), Jiangsu (n=90,710), Zhejiang (n=72,212), Shandong (n=68,065), and Beijing (n=66,057). The proportion of daily online MSM among adult men in different cities varied from 0.04% to 0.96%, with a mean of 0.20% (SD 0.14%). Three migrating centers—Guangdong, Beijing, and the Yangtze River Delta (Shanghai-Zhejiang-Jiangsu)—accounted for 57.23% of MSM migrants in the county.

**Conclusions:**

The percentage of MSM among adult men in mainland China is at the middle level compared with other Asia and Pacific countries. However, the number of MSM is very large, and the distribution is uneven. Both MSM distribution and migration are highly affected by socioeconomic status.

## Introduction

Men who have sex with men (MSM) have a substantially disproportionate risk for HIV infection, and the number of HIV cases found among MSM continues to increase in most countries [1-3]. In the United States, MSM accounted for 53% and 67% of new HIV diagnoses in 2006 and 2014, respectively [4,5]. In China, the risk of HIV transmission among MSM is estimated to be 45 times higher than that of heterosexual transmission [6]. The prevalence of HIV in MSM sentinel surveillance continues to increase in China, with rates ranging from 0.9% to 8.0% between 2003 and 2015 [7-9]. In 2006, MSM accounted for only 2.5% of the newly diagnosed HIV/AIDS cases in China, whereas that number was 25.5% in 2017 [10]. To monitor the prevalence of HIV among MSM, China has built 108 national sentinel sites [11]. The sentinel surveillance collects and reports HIV testing data among MSM and the characteristics of the target population by sampling, although they do not collect data on the total number of MSM. The population size of MSM is an important number for calculating various disease rates among MSM. Without the population-level denominators for MSM, it is difficult to accurately describe the burden disparity of HIV among MSM in different regions.

Purcell et al [5] estimated the proportion of men aged 13 years and older in the United States who engaged in same-sex behavior to be approximately 3.9%. By combing multiple data sources, Grey et al [12] developed refined estimates of the size of the MSM population at the county, city, and state levels. Many studies have estimated the population number by surveying MSM with social networking and mobile phone apps (eg, Grindr [13], Jack’d [14,15], Hornet [16], and Blued [17-19]). Using social app technology and internet-based surveys, MSM numbers in two regions of Vietnam (Ho Chi Minh City and Nghe An province) were estimated. The proportions of MSM among adult men in these regions were 1.35% and 0.17%, respectively [20]. Algarin et al [21] examined the spatial distribution of geosocial networking app usage of MSM in Fayette County, a midsize city in the southern United States, and found that user density was highest in areas with higher populations, lower incomes, and more businesses. In China, based on population sampling, some studies suggest that MSM account for 2% to 4% of adult men [22,23]. The MSM population in several other cities has been estimated in many studies, including Beijing [24], Wuhan [25], Shanghai [26], Chongqing [27], and Shenzhen [28]. Despite the growing significance of MSM in the HIV epidemic and efforts in estimating HIV burden, nationwide figures, including population size, geographical distribution, and migration, are still largely unknown in China. Social media big data in China may help fill this gap.

China has experienced dramatic development in information technology in the past two decades. At the end of 2015, 85.3% of young people had access to the internet, 90% of them via mobile phones [29]. Technological development has significantly changed the ways of socializing and seeking same-sex partners for the MSM population. The internet and mobile social networking apps have largely replaced traditional meeting places, such as bathhouses and bars, and they can be conveniently used to socialize and anonymously seek nearby partners in real time, avoiding MSM stigma and harsh local sociocultural environments [30,31]. In this study, we use big data from social networking to estimate the number, spatial distribution, and migration of MSM populations in mainland China.

## Methods

### MSM Social Networking Big Data

In mainland China, the mobile phone app Blued is the most popular app dedicated to MSM social networking [32]. The app was developed in 2012 by Blued International Inc, a company founded in 2000 [33]. The app has a market share of more than 90% for MSM communication apps in China [34]. Blued allows users to create personal profiles with pictures and personal information, including age, height, and weight. Like other similar social communication apps, such as Jack’d and Hornet, Blued displays nearby users and their profiles [33]. By simulating moving mobile phone positions along with a regular grid covering the whole country, we collected 85 days of data on online users across China from January 7 to March 28, 2018, and October 22 to November 4, 2018. The data-collecting process contained the following steps. First, we created a 5-kilometer regular grid covering the whole of mainland China. Grids with no people living in them were removed based on the 2015 annual Visible Infrared Imaging Radiometer Suite (VIIRS) nighttime light [35]. Second, to simulate position changing, we installed Blued on an android emulator, NoxPlayer, which can run on a computer [36]. The emulator enables users to set a virtual location. Third, by changing the virtual location of the emulator with coordinates of a grid center and then refreshing Blued, the nearby list on Blued could be updated. We recorded the list and repeated the step. After that, we counted the number of users in each grid and removed duplicated records. To accelerate the operation, an auxiliary computer language program was also developed to automate the process.

In the study, the spatial distributions of MSM social networking users in two periods were compared to find out how users were migrating within the country during the Spring Festival holidays from February 16 to 20, 2018, and during nonholidays (ie, after the Spring Festival travel season from March 12 to 28, 2018). The day of February 21, 2018, was excluded from the holidays because of the large number of people returning to work on the last day of the holidays. For nonholidays, we only used the data after Spring Festival travel because most people have usually returned to work, and the population distribution is more stable. The distribution of MSM during the nonholiday period was considered as the regular MSM population pattern, whereas the distribution of MSM during the holiday period was considered as the origin where the MSM population came from because most Chinese people reunite with their families during the traditional Lunar New Year holidays [37,38].

The Gini coefficient was adopted to measure whether MSM were equally distributed among adult men [39]. It ranges from 0 to 1, with 0 representing perfect equality and 1 representing perfect inequality. Spatial pattern similarity between two geographic distributions was measured by Geodetector q statistic [40].

### MSM Population Estimation in China

The obtained MSM data were from only a portion of the total social networking MSM population in China. We designed an experiment based on the capture-mark-recapture method to estimate the overall social networking MSM population. Capture-mark-recapture is an efficient method, originating from biological science, to estimate the size of an animal population [41]. The method requires at least two independent capture samples. For the first sample, a number of animals are captured (sample size *m*), marked, and released back into the population. Then, in the second sample (sample size *n*), recaptured animals can be identified (overlapping sample size *k*). If the two samples are independent, and each animal has an equal capture possibility, then the case number can be estimated by (*m*+1) * (*n*+1)/(*k*+1)−1 for a closed population [41,42]. The capture-mark-recapture method is widely used in a variety of applications, including biology, ecology, and epidemiology [42-44]. We designed an experiment consisting of two independent samples, with each sample covering a period of two weeks, to record online social networking MSM on the Blued app. The first sampling was conducted from March 12 to 28, 2018, and the second sampling was performed from October 22 to November 4, 2018. The time interval between sampling periods was approximately seven months. The total provincial number of social networking MSM was then estimated using the capture-mark-recapture method. However, some MSM do not use social networks. Therefore, we estimated the total provincial MSM population by dividing the provincial social networking MSM population by provincial internet penetration, reported in the China Internet Development Report in 2017 [45].

### Ethical Statement

The study only collected public data. No private information was collected, stored, analyzed, or published; thus, no ethical approval and patient consent were required.

## Results

### Provincial Spatiotemporal Heterogeneity of MSM Using the Social Networking App

**Figure 1:**
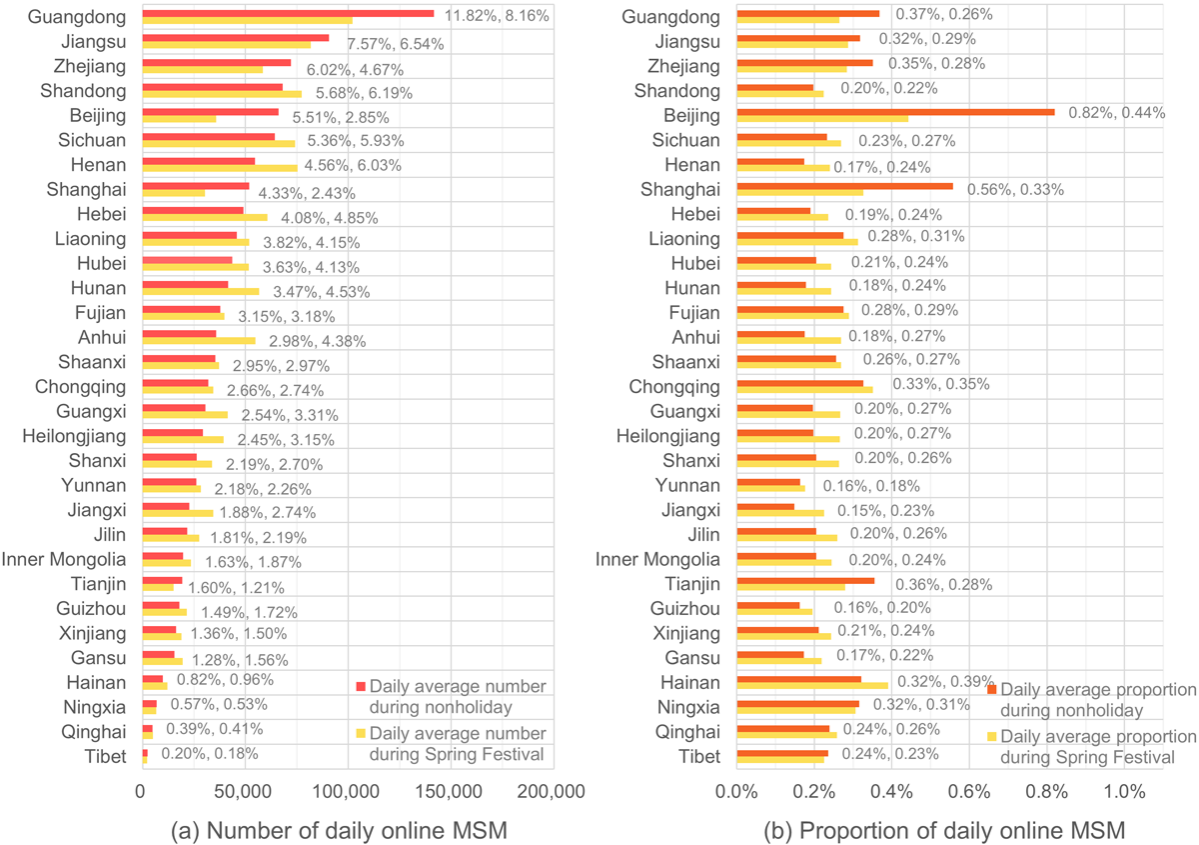
Provincial daily number and proportion of social networking men who have sex with men (MSM) from the Blued app. Numbers after the bars in (a) denote the percent of daily online MSM among the whole country; numbers after the bars in (b) denote the proportion of daily online MSM among local adult men aged 18 to 64 years; the two numbers after each bar are for nonholiday and holiday respectively.

Data on 3,429,705 social networking MSM in China were collected during the study period from January 7 to March 28, 2018. Among these MSM, 2,010,096 and 2,449,493 were online during the Spring Festival holidays (February 16-20) and nonholidays (after the Spring Festival travel season from March 12-28), respectively. During nonholidays, the average number of daily online MSM was 1,198,682 across mainland China. The 10 provinces (including municipalities) with the highest average number of daily online MSM numbers were Guangdong (n=141,712), Jiangsu (n=90,710), Zhejiang (n=72,212), Shandong (n=68,065), Beijing (n=66,057), Sichuan (n=64,258), Henan (n=54,642), Shanghai (n=51,908), Hebei (n=48,926), and Liaoning (n=45,773). These provinces accounted for 58.75% of the number of daily online MSM in mainland China (Figure 1). The number of social networking MSM in Guangdong, which accounted for 11.82% of daily online MSM across all mainland China during the nonholiday period, was higher than that in other provinces. Qinghai and Tibet provinces had fewer than 5000 daily online MSM. Ningxia and Hainan provinces had fewer than 10,000 daily online MSM. By dividing the number of daily online MSM by the number of adult men aged between 18 and 64 years (from the 2010 national population census), Beijing and Shanghai were found to be the two municipalities with the highest proportions of daily online MSM (0.82% and 0.56%, respectively), followed by Guangdong (0.37%), Tianjin (0.36%), Zhejiang (0.35%), Chongqing (0.33%), Jiangsu (0.32%), Hainan (0.32%), and Ningxia (0.32%) (Figure 1).

**Figure 2:**
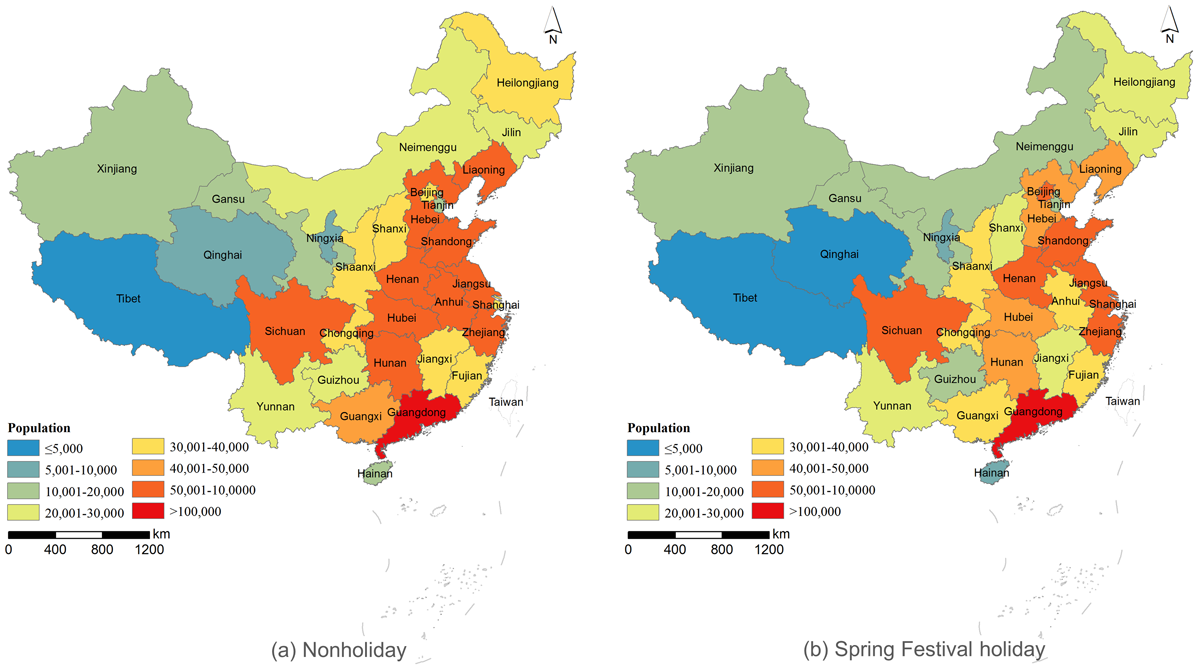
Spatial distribution patterns of daily online MSM from the Blued app.

The average numbers of daily social networking MSM were similar during nonholidays and the Spring Festival holidays (1,198,682 and 1,250,676, respectively). However, the spatial pattern during the Spring Festival differed slightly from that during the nonholiday period (Figure 2). The similarity of the two spatial patterns was 0.85 (*P*<.001), as measured by the Geodetector q statistic [40]. The 10 provinces or municipalities with the highest average number of daily online MSM during the holidays were Guangdong (n=102,035), Jiangsu (n=81,816), Shandong (n=77,416), Henan (n=75,366), Sichuan (n=74,161), Hebei (n=60,675), Zhejiang (n=58,442), Hunan (n=56,709), Anhui (n=54,751), and Liaoning (n=51,850) as seen in Figure 1. In terms of the proportion of daily online MSM, Beijing was the highest (0.44%), followed by Hainan (0.39%), Chongqing (0.35%), Shanghai (0.33%), Liaoning (0.31%), and Ningxia (0.31%), as seen in Figure 1.

The Pearson correlation coefficient between the number of provincial social networking MSM and provincial gross domestic product in 2018 was .92, which is higher than the Pearson correlation coefficient between the number of provincial social networking MSM and the number of provincial adult men (*r*=.81). The Gini coefficients of provincial daily active MSM on nonholidays (March 12-28) and the Spring Festival holidays (February 16-20) were 0.39 and 0.34, respectively, indicating that the distribution of daily online MSM during holidays was more evenly distributed than during nonholidays because of online MSM living in cities going home. The difference in daily online MSM during nonholidays and the Spring Festival holidays was positive in eight provinces and negative in 23 provinces. The four provinces with positive surplus values greater than 10,000 were (in descending order) Guangdong, Beijing, Shanghai, and Zhejiang. Seven provinces had a negative surplus of less than −10,000: Henan, Anhui, Hunan, Hebei, Jiangxi, Guangxi, and Heilongjiang (in ascending order).

### Spatiotemporal Variation of City-Level Daily Social Networking MSM

**Figure 3:**
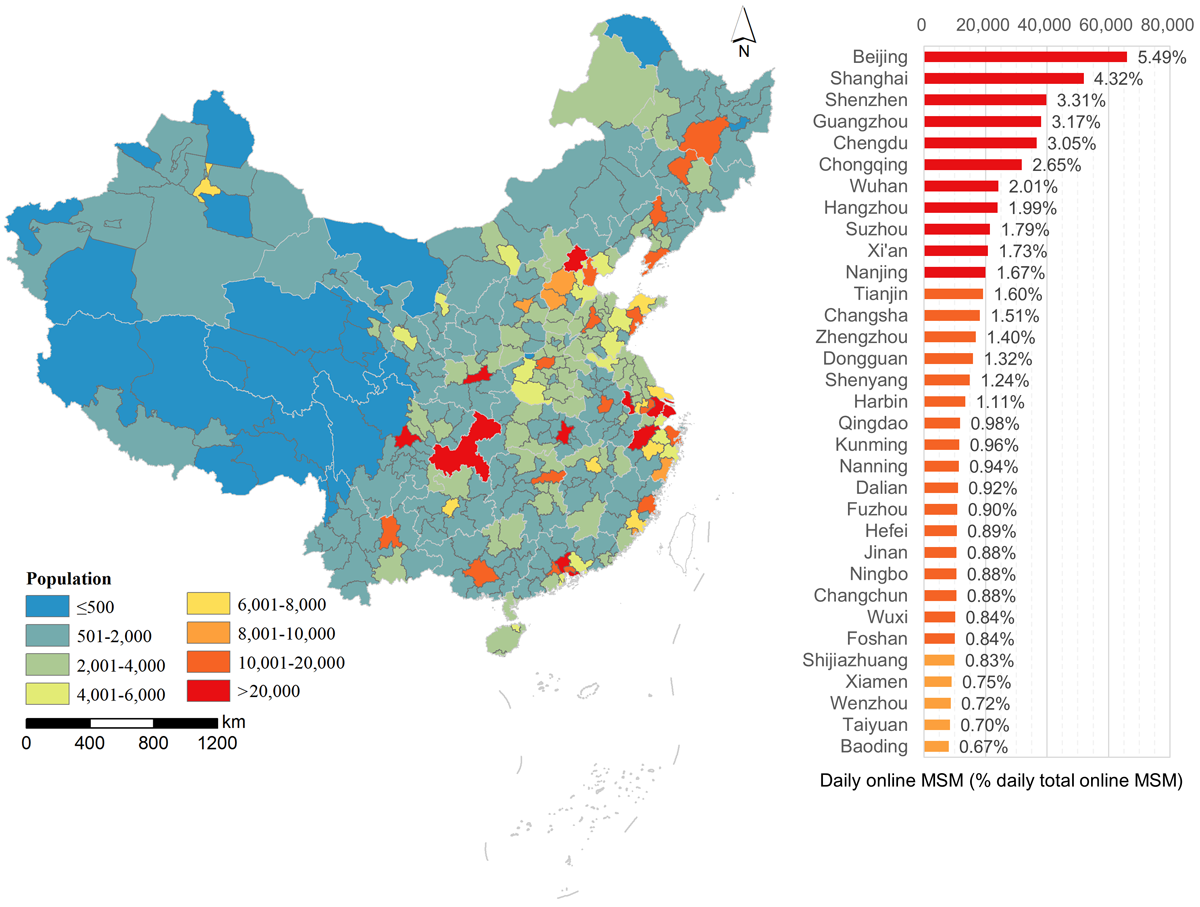
City-level distribution of daily online MSM from nonholiday social networking.

The number of daily online MSM varied greatly between different prefecture-level cities (Figure 3). Among 344 prefecture-level cities, 50.23% of nonholiday daily online MSM were in only 29 cities, whereas 28 cities had daily online MSM numbers greater than 10,000. In 11 cities (Beijing, Shanghai, Shenzhen, Guangzhou, Chengdu, Chongqing, Wuhan, Hangzhou, Suzhou, Xi’an, and Nanjing, in descending order), the number of daily online MSM exceeded 20,000. These cities accounted for 31.26% of daily online MSM in the country. The variation in social networking MSM numbers during the Spring Festival holidays was smaller than that during nonholidays: the coefficients of variation were 1.15 and 1.93, respectively. During the Spring Festival holidays, Chongqing had the second largest number of online MSM; Beijing and Shanghai had the largest and third-largest numbers of online MSM, respectively, and Chengdu replaced Guangzhou as having the fourth-largest number of online MSM.

**Figure 4:**
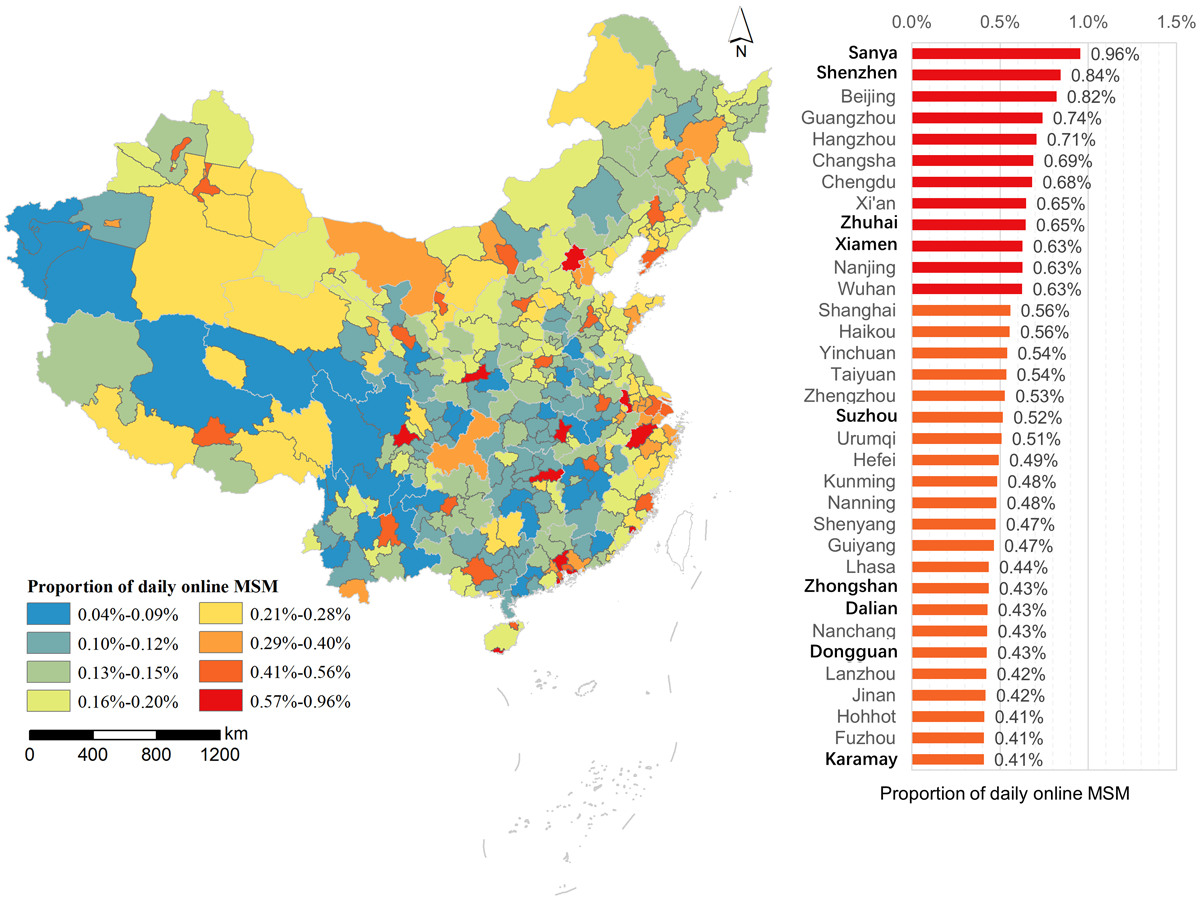
City-level distribution of the proportion of daily online MSM from nonholiday social networking. Cities in bold type are nonprovincial capitals.

The proportion of daily online MSM among adult men in different cities varied from 0.04% to 0.96%, with a mean of 0.20% and a standard deviation of 0.14% (Figure 4). Although the general distribution of high proportions of daily online MSM was similar to the distribution of high numbers of daily online MSM, the order of cities changed significantly. The top 10 cities, from high to low proportion, were Sanya, Shenzhen, Beijing, Guangzhou, Hangzhou, Changsha, Chengdu, Xi’an, Zhuhai, and Xiamen. Most of these cities are provincial capitals.

### Spatiotemporal Pattern of Social Networking MSM Migration Flow

**Figure 5:**
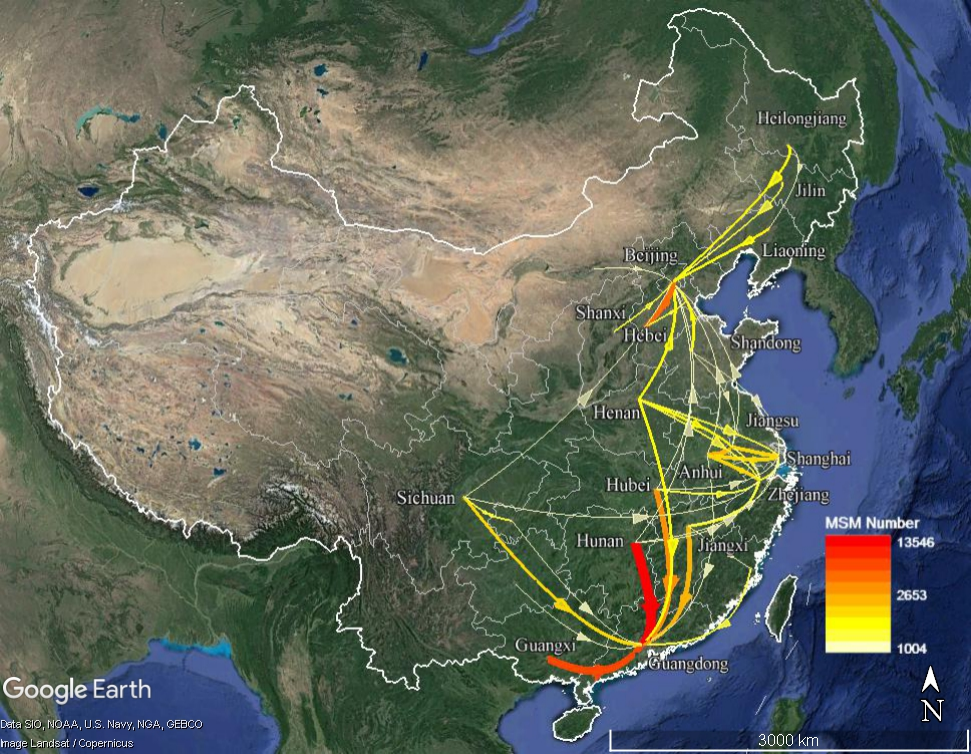
Interprovincial MSM migration flows (n≥1500) from the social networking data.

A total of 1,705,456 MSM were online during both the Spring Festival holidays and the nonholidays. Origin-destination analysis based on co-occurring social networking MSM in the two periods showed that 391,915 social networking MSM migrated between provinces, accounting for approximately 22.98% of all social networking MSM. From the social networking MSM data, the top five MSM destinations were Guangdong, Beijing, Shanghai, Zhejiang, and Jiangsu (Figure 5), which accounted for 57.23% of migrants in mainland China. For Guangdong province, three provinces were the source of more than 10% of MSM migrants each: Hunan (19.05%), Guangxi (16.10%), and Hubei (10.70%). Jiangxi province was another important source province of MSM migrants for Guangdong, accounting for 9.36%. For Beijing, Hebei was the largest source of MSM migrants, accounting for 18.20%, which is much more than the second- and third-largest sources, Shandong (8.73%) and Henan (7.10%). Jiangsu and Anhui were the top two MSM migrant source provinces for Shanghai, accounting for 16.08% and 15.04%, respectively. The MSM migrants in Zhejiang were from three main source provinces, each accounting for more than 10% of MSM migrants: Anhui (17.79%), Jiangxi (12.53), and Henan (10.15%). Furthermore, Anhui (23.54%) and Henan (12.67%) were the two largest MSM migrant sources for Jiangsu province.

### Total Number of MSM in China

**Figure 6:**
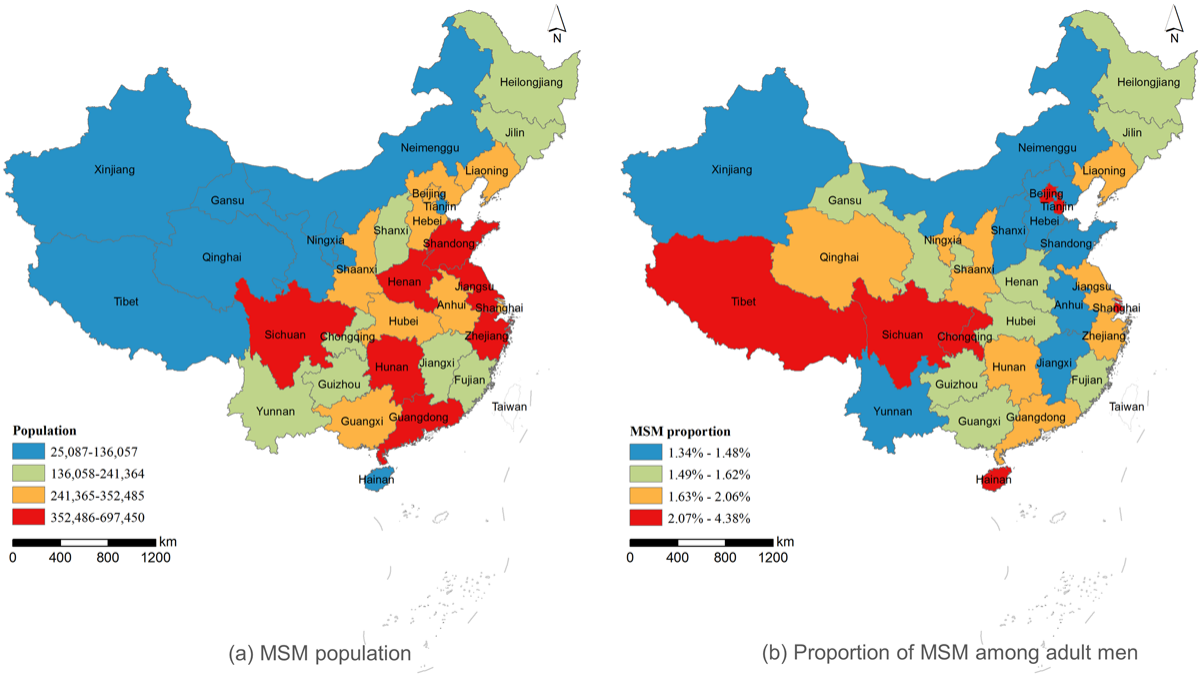
Spatial distribution of estimated MSM number (a) and proportion (b) among adult men in mainland China.

We recorded the profile IDs of 2,449,493 social networking MSM captured in the first sample period from March 12 to 28, 2018, and 2,474,293 social networking MSM in the second sampling from October 22 to November 4, 2018. According to the profile IDs from the two samples, 1,332,632 MSM were recaptured in the second sampling. The provincial numbers of social networking MSM were estimated by the capture-mark-recapture method and then adjusted by internet penetration to obtain the total number (Table 1). The sum of all provincial MSM number estimates was 8,288,536 (95% CI 8,274,931-8,302,141) in mainland China, accounting for approximately 1.732% (95% CI 1.729%-1.734%) of adult men aged between 18 and 64 years in China. The provincial number of MSM varied from 25,087 to 697,450, with a mean of 267,372 and a standard deviation of 163,061. The ratio of MSM to adult men also varied greatly by province, from 1.34% to 4.38%, with a mean of 1.85% and a standard deviation of 0.61%. The results are shown with quantile breaks in Figure 6. Seven provinces fell into the fourth quantile of MSM number, the highest level in Figure 6: Guangdong, Sichuan, Jiangsu, Henan, Shandong, Zhejiang, and Hunan (in descending order). For the ratio of MSM to adult men, the seven top provinces falling into the fourth quantile interval were Beijing, Shanghai, Tibet, Chongqing, Hainan, Tianjin, and Sichuan (in descending order). Sichuan was the only province in the fourth quantile with a large number of MSM and a high ratio, whereas Xinjiang and Inner Mongolia, in the first quantile, had both a small number of MSM and a low ratio. The similarity of the two spatial patterns was only 0.37 (*P*<.001), as measured by the Geodetector q statistic. This indicates that there was a significant difference between the spatial distribution of the MSM population and the proportion of MSM among adult men.

**Table 1:** Estimated provincial numbers and proportions of men who have sex with men (MSM) among adult men in mainland China.

Province	Internet penetration,^a^ %	Social networking MSM, n	MSM, n (% of adult men)
Guangdong	74.0	516,113	697,450 (1.81)
Sichuan	43.6	248,679	570,365 (2.07)
Jiangsu	56.6	317,627	561,178 (1.97)
Henan	43.4	208,489	480,389 (1.53)
Shandong	52.9	249,935	472,467 (1.37)
Zhejiang	65.6	268,375	409,108 (1.99)
Hunan	44.4	174,478	392,968 (1.69)
Beijing	77.8	274,233	352,485 (4.38)
Hebei	53.3	184,049	345,308 (1.34)
Hubei	51.4	168,538	327,895 (1.55)
Anhui	44.3	131,758	297,422 (1.46)
Liaoning	62.6	176,804	282,435 (1.70)
Shanghai	74.1	204,844	276,443 (2.97)
Shaanxi	52.4	131,830	251,584 (1.82)
Guangxi	46.1	112,368	243,748 (1.57)
Chongqing	51.6	124,544	241,364 (2.47)
Yunnan	39.9	94,461	236,744 (1.48)
Heilongjiang	48.1	111,801	232,435 (1.57)
Jiangxi	44.6	96,776	216,987 (1.43)
Fujian	69.7	148,229	212,667 (1.55)
Shanxi	55.5	95,710	172,450 (1.35)
Jilin	50.9	87,452	171,811 (1.62)
Guizhou	43.2	71,265	164,965 (1.50)
Inner Mongolia	52.2	71,022	136,057 (1.42)
Gansu	42.4	57,249	135,021 (1.52)
Tianjin	64.6	77,784	120,409 (2.23)
Xinjiang	54.9	59,678	108,703 (1.41)
Hainan	51.6	39,051	75,680 (2.46)
Ningxia	50.7	22,611	44,598 (2.06)
Qinghai	54.5	17,610	32,312 (1.64)
Tibet	46.1	11,565	25,087 (2.49)

^a^Internet penetration was from the 39th China Internet Development Report [45].

## Discussion

Compared with other members of the population, MSM have a significantly higher risk for HIV infection [1-3]. The MSM population and distribution are critical parameters for precision prevention of the deadly disease, to realize the goal of ending the AIDS epidemic by 2030 and to achieve the Joint United Nations Programme on HIV/AIDS (UNAIDS) 90-90-90 targets [46,47]. Our estimates are the first comprehensive estimation of the MSM population size, geographical distribution, and migration conducted in mainland China. These results will be an important contribution to accurately carrying out intervention for high-risk groups, expanding testing, and realizing the first 90% in China. For example, from the research result, we can identify provinces and cities with large MSM numbers, such as Guangdong, Sichuan, Jiangsu, Henan, Shandong, Zhejiang, and Hunan, which should have more resources allocated to them.

There is considerable spatiotemporal heterogeneity for social networking MSM in mainland China. The difference in the spatial distribution patterns of daily online MSM during nonholidays and the Spring Festival holidays might indicate that MSM are distributed rather evenly among all adult men. However, urbanization and economic development might be positive drivers of MSM accumulation. From the provincial social networking MSM distribution in Figure 2, it can be seen that provinces with large numbers of social networking MSM are mainly located in eastern China, and they usually have a large population and high gross domestic product. Thus, economic development level might be an important factor for the distribution of social networking MSM. Large or well-developed cities tend to have more social networking MSM because cities often have better network facilities and higher internet penetration. Provincial migration direction of MSM was from less-developed provinces to more-developed provinces, such as Guangdong, Beijing, Shanghai, Zhejiang, and Jiangsu. The social networking MSM migration flow was generally consistent with population migration flow in mainland China [37]. In recent decades, fast urbanization in China has brought large numbers of rural laborers flocking to cities to find jobs, which might increase the chance to use mobile apps to seek nearby friends for MSM coming from less-developed regions.

The study reveals that mainland China has approximately 8,288,536 MSM, approximately 1.8 times the number in the United States, although the percentage of MSM among adult men in mainland China (1.73%) is lower than that in the United States (3.9%) [12]. Our estimate of the percentage of MSM among adult men is also lower than the previous estimates of 2% to 4% in China [22,23]. The difference may come from different sampling designs. In traditional sampling-based studies to estimate the MSM population, the sample size was often limited to hundreds or thousands. Without enough a priori information on MSM distribution, it is hard to design an unbiased sampling to cover a country as large as China. Our estimates made use of social networking data, which have no sampling size limit and avoid potential bias due to sampling design. The estimate shows that 10 provinces have a percentage equal to or greater than 2%. More than one-half (51.66%) of the total MSM population reside in only nine provinces. The phenomenon of uneven distribution of MSM in mainland China is consistent with the distribution in the United States, where more than one-half of the total US MSM population is located in only seven states [12]. The estimated percentage of MSM among adult males is at the middle level compared with the range of 0.09% to 4.06% for Asia and Pacific countries [48].

The internet and mobile geosocial networking information technology have become not only the major tools for socializing and seeking same-sex partners for MSM populations in recent years but they can also help with surveillance of the risk [30,31]. Compared with traditional offline approaches, online sex-seeking might lead to multiple sexual partners, a higher probability of having unprotected sex, and a higher possibility of being diagnosed with sexually transmitted infections, which is inevitably associated with a higher risk of HIV/AIDS infection [30,49]. However, online approaches can be used as an important window for health-related organizations to spread health protection messages, such as the location of the nearest testing center for sexually transmitted infections [31]. Online approaches should be used to help to control the incidence of HIV/AIDS for MSM in China.

To estimate the number of MSM in China, we had to make several assumptions that might limit the interpretation of our results. First, we assumed that the popular Blued app accounts for most social networking users in mainland China, but the proportion of MSM users might vary slightly across China. Second, we ignored population movement between the two samples in the capture-mark-recapture experiment, which might violate the closed population assumption. However, the total population movement would be small compared with the provincial population. Third, in the migration analysis, we did not consider that there might be a small portion of MSM who traveled to other locations as tourists during the Spring Festival. Although some limitations are associated with our results, we believe the results do provide a lower estimate of the MSM population in mainland China [50].

The study presents a promising and efficient method for estimating MSM populations in different areas of mainland China for the first time. We found that there are more than 8 billion MSM in mainland China, accounting for approximately 1.73% of adult men aged 18 to 64 years. The MSM population is unevenly distributed in different cities and provinces. The spatiotemporal distributions of MSM revealed by this study provide new opportunities for determining the burden of HIV and sexually transmitted infections among MSM in different areas. The nationwide estimates of MSM populations and geographical distributions could provide public health practitioners and policymakers with a useful tool for better resource allocation, intervention development, and service delivery. Provinces and cities with large MSM numbers and high proportions of MSM should be considered for allocation of more resources to control the potential HIV/AIDS risk.

## Abbreviations

MSM: men who have sex with men
